# A Continuous Labour Supply Model in Microsimulation: A Life-Cycle Modelling Approach with Heterogeneity and Uncertainty Extension

**DOI:** 10.1371/journal.pone.0111903

**Published:** 2014-11-12

**Authors:** Jinjing Li, Denisa Maria Sologon

**Affiliations:** 1 Institute of Governance and Policy Analysis, University of Canberra, Canberra, Australia; 2 Maastricht University/UNU-MERIT, Maastricht, the Netherlands; 3 CEPS/INSTEAD, Esch-sur-Alzette, Luxembourg; 4 Institute for the Study of Labor (IZA), Bonn, Germany; Universidad Veracruzana, Mexico

## Abstract

This paper advances a structural inter-temporal model of labour supply that is able to simulate the dynamics of labour supply in a continuous setting and addresses two main drawbacks of most existing models. The first limitation is the inability to incorporate individual heterogeneity as every agent is sharing the same parameters of the utility function. The second one is the strong assumption that individuals make decisions in a world of perfect certainty. Essentially, this paper offers an extension of marginal-utility-of-wealth-constant labour supply functions known as “Frisch functions” under certainty and uncertainty with homogenous and heterogeneous preferences. The lifetime models based on the fixed effect vector decomposition yield the most stable simulation results, under both certain and uncertain future wage assumptions. Due to its improved accuracy and stability, this lifetime labour supply model is particularly suitable for enhancing the performance of the life cycle simulation models, thus providing a better reference for policymaking.

## Introduction

The empirical literature on structural labour supply models has gained an increasing interest over the past decades, as these models became important tools for policy makers to examine potential reform options. The continuous approach in the tradition [1] has been complemented by an approach based on a discrete choice specification, mainly inspired by [2], [3]. The arbitrary discretisation of the working hours, however, can lead to a loss of information in the estimation, which can produce misleading results with inappropriate groupings. Hybrid approaches also exist, for example [4], where the male decision is discrete while the female decision is continuous. This paper examines the uses of continuous labour supply models in the context of forward simulations and explores the potential of incorporating better controls for heterogeneities in a microsimulation context.

The original interest in life-cycle labour supply was motivated by the need to investigate various dimensions of labour supply, such as the determinants of the shape of the life-cycle hours profile, the labour supply response to the aggregate wage, the changes and the source of the idiosyncratic year-to-year changes in labour supply. The existing literature, however, manages to shed little light on the original questions. It focuses mainly on one aspect of the inter-temporal hours variation, namely the labour supply response to the wage growth along a known life-cycle trajectory, whilst ignoring other aspects. One ignored aspect is the labour supply response to wage changes under uncertainty, meaning wage changes that determine individuals to revise their expectations of their future wages [5].

The life-cycle framework is proposed as an explanation for all components of the individual labour supply [5]. The life-cycle model can be used to explain the aggregate year-to-year movements in labour supply “time effects” [6], the systematic age effects in hours of work (“age effects”) and the differences across people with respect to their hours of work over the life-cycle “person-specific effects”. With the integration the labour market behaviours, the life-cycle framework can also shed light on behaviours that span multiple phases in a person's life, such as consumption smoothing and borrowing [7], [8].

In the context of estimating and simulating life-cycle labour supply, the choice of the labour supply elasticity to be simulated depends on the goal. If the goal is to compare the impact of wage variations across consumers on labour supply, the variation in the entire wage profile must be examined. Because the variation of the wage profile affects the value of the marginal utility of wealth, the Frisch elasticity cannot be used. Estimating the full impact on wages requires estimating the effect of shifts of the wage profile on hours of work besides the estimation of the inter-temporal elasticity. The estimation of the full impact of wage changes, both evolutionary and parametric, are of core importance for policy evaluation. Assuming that tax and benefit reforms represent unanticipated shifts in net real wages today and in the future, the elasticity measuring the cumulated response to evolutionary changes and parametric shifts in the life-cycle wage profile is the most appropriate for describing the response to these reforms [9].

Given the complex nature of the human economic behaviours, it is unsurprising to observe a large degree of population heterogeneity. One method to incorporate individual heterogeneity in the model is to estimate individual specific effect, which can be estimated either as fixed or random effects. The choice between fixed and random effects relies mainly on whether the individual specific effects can be assumed independent of the explanatory variables included in the model. Assuming that the individual component is correlated with the explanatory variables triggers many problems in a simulation. The estimated coefficients cannot be used to generate a conditional prediction of individual earnings without specifying the joint process determining the individual specific effects and the explanatory variables [10], [11]. The impracticality of this option, together with the fact that the fixed-effect specification cannot accommodate covariates that are constant over time, constrained most studies to maintain the assumption of a zero correlation between the individual specific effects and the other covariates, a rather strong and improbable assumption. The main drawback of error components models is they provide less stable simulations due to the stochastic components, which affect the reproducibility of the results.

Another way to incorporate the heterogeneity effects is to use random coefficient models. There are discussions on implementing the random coefficient models in discrete models, usually in the form of mixed logit, e.g., [12], [13], but the discussions on the continuous model are limited. Provided that heterogeneity is present in empirical models of labour supply, the application of random coefficients models is necessary to avoid biased estimates. The main drawback of these models, however, is their high computational cost. Given this limitation, existing studies suggest that if heterogeneity is non-existent or the bias is insignificant, the standard fixed coefficient models represent the optimal choice [14].

This paper proposes a structural inter-temporal model of labour supply, which estimates and predicts the dynamics of labour supply in a continuous setting. It aims to capture the individual heterogeneity to a larger extent than the existing labour supply models while maintaining the consistency with the lifetime economic theory. The model is estimated using both a transformed fixed effect specification that circumvents the standard problems mentioned above, and a random coefficient specification. Additionally, the model incorporates uncertainties regarding future wages to further explore heterogeneity.

## A Life-Cycle Model of Labour Supply

Our model follows the theoretical model introduced by [15], [16] and [17], and the unifying labour supply framework introduced by [9]. The model aims to estimate the effect of evolutionary wage changes assuming no parametric shift in the wage profiles. The labour supply responses are estimated and simulated under two scenarios: (i) people make decisions in a world of perfect certainty; (ii) people are uncertain about their future wages.

### A Life-Cycle Model of Labour Supply under Certainty

Under the assumption of certainty, the effect of evolutionary wage changes on hours of work represents the conventional “marginal-utility-of-wealth constant” inter-temporal elasticity of labour supply obtained from the Frisch labour supply equations and the Euler condition. The marginal-utility-of-wealth parameter serves as a sufficient statistic that captures the information from the other periods needed to solve the maximization process in the current period [9].

The inter-temporal elasticity estimated from the Frisch specifications is relevant for predicting the individual labour supply into the future assuming a steady state, mainly due to the presence of the marginal utility of wealth, which is individualized, constant over time, and accounts for the worker's future plans [18].

Following [16], [19], the theory underlying our model of lifetime hours of work is an extension of permanent income theory [20] to a situation where the relative price of consumption and leisure varies over the life-cycle. The permanent income hypothesis can be extended to lifetime labour supply to assume that individuals/households look into the future when deciding the current number of hours supplied on the labour market. This theory allows the distinction between a consumer's dynamic behaviour and the factors determining the differences in hours worked between consumers. This separation leads to a manageable empirical model that accommodates the differentiation between labour supply responses to evolutionary changes and those to parametric changes in the wage profiles.

#### An economic model of labour supply under certainty with homogenous preferences and heterogeneous individual effects

We start with an economic model of life-cycle labour supply decisions assuming that workers make decisions regarding future income in an environment of perfect certainty. Workers are assumed to choose consumption and hours of work at each age to maximize a lifetime preference function, strongly separable over time, subject to wealth constraints. The model described below is designed for single decision-makers, but an extension to joint decision makers is straightforward.

Men's labour supply behaviour is assumed independent, while women's labour supply is conditioned on other household incomes besides their own earnings. Assuming that consumer *i* at age *t* has a utility given by the concave function 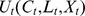, where 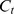 is consumption at age *t*, 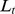 is the number of hours of leisure at age *t* and 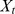 is a vector of “taste shifters” at age *t*, 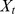 can include both observed and unobserved variables. Due to the assumption of separating utility, the lifetime preference function is the sum of discounted future utilities at moment 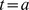 (the beginning of the active life), where *t* is age and 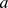 the age of entering the labour market:
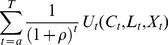
(1)


The lifetime (active life) is assumed to consist of 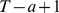 periods, where *T* is retirement age. 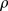 is rate of time preference used for discounting the value of future utility. Formally, the consumer has to choose 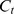 and 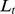 at each age to maximize their lifetime preference equation (1) subject to a lifetime wealth constraint.
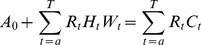




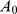
 represents the assets at the start of the active life, 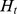 hours worked at age *t*, 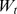 the exogenous wage rate at age t, and 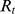 the discount rate. In period *t*, the consumer can borrow/lend at a constant interest rate 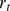. To create the Frisch labour supply functions, we assume the contemporaneous individual utility function at age *t* is:
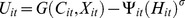




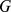
 is a monotonically increasing function of 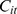, 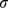 is a time-invariant preference parameter common across consumers. 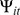 is an age-specific “tastes” parameter which depends on the characteristics which influence the utility at age *t* via 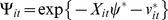, where 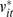 is the contribution of unobserved characteristics and 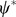
is a vector of preference parameters. The preference parameter includes the participation decision. Assuming an interior optimum, the implied Frisch labour supply function or marginal utility of wealth constant labour supply function is obtained from maximizing the utility in period *t* subject to the lifetime wealth constraint.

The first order conditions of the Lagrange function result in the Frisch or “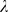 constant” consumption and labour supply functions. For hours worked at age *t*, this implies:
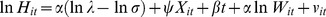
(2)

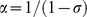
, 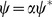, 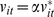, and 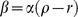 are assumed constant across consumers and time. Equation (2) is the Frisch or marginal utility of wealth constant labour hours of work function if the individual chooses to work. It decomposes the labour supply decision regarding hours worked into time *t*, personal characteristics 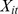, the wage rate 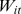 and the 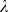 component, which is the sufficient statistic summarizing the information for each consumer from the other periods. The optimal value of 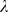 is obtained by substituting the 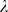 constant consumption and labour supply functions into the budget constraint. 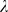 is a function of initial assets, lifetime wages, interest rates, rates of time preference and tastes. 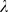 is the correspondent statistic to the permanent income from the permanent income theory in [20], thus a permanent component, which together with current wage determines the consumer's current consumption and labour supply. 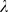 also incorporates historic and future information on lifetime wages and assets that are relevant for current consumption and labour supply choices. The conclusion is that, assuming certainty, the “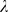 constant” consumption and labour supply functions fully characterize a consumer's dynamic behaviour [9], [15], [18].

As shown by [9], in a world of perfect certainty, 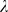 can be captured as an individual specific effect which is constant over time, thus the changes in individual wages have no impact on 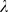. Following [9], [18], we approximate 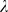 by: 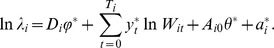




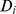
 is a vector of observed time-invariant or rarely changing characteristics, 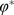, 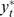, 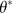 are parameters assumed constant across consumers, and 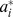 is an error term. This specification imposes strong restrictions, as it assumes each worker knows the number of years he will work 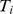, and total lifetime income. It also incorporates the effect of interest rates and rates of time preference in the intercepts and the other parameters. The time invariant characteristics 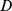 can therefore be decomposed into an observed part 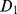 and an unobserved part, 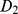. The observed part can be approximated by a linear form of 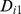, whereas the unobserved part, assumed to follow a normal distribution, is included in the error term. Thus, the individual unit effect can be estimated by a linear function of time-invariant characteristics and a normally distributed error that captures the unexplained individual effect. Therefore, we can rewrite equation (2) as:
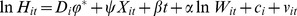



The mechanism for predicting lifetime wage profiles is approximated by:
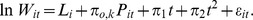




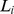
 is the individual-specific effect, 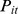 is a vector of personal and professional career characteristics, 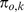 is a vector of coefficients (time and individual invariant), 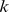 is the number of personal and professional career characteristics and 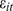 the error term.

#### An expansion of the economic labour supply model under certainty with heterogeneous preferences

Next, the model is extended by allowing for increased individual heterogeneity in labour supply preferences. The preference parameter 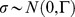 is individual-specific and time-invariant. 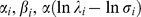 are assumed to be individual-specific and follow normal distributions across individuals. The lifetime labour supply function becomes:
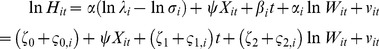
(3)where 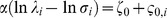 is the individual effect (time-invariant), and 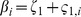 and 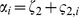 are the random coefficients of age and wage. 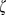 is the mean intercept of hours of work, 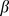 the mean slope of age, 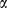 the mean slope of ln(wage) and 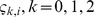 the deviation of individual intercepts and slopes from the mean values of 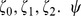 is a vector of fixed coefficients, individual and time invariant. We can also allow for increased heterogeneities in the wage estimations using the similar random coefficient approach.

#### An expansion of the economic labour supply model under uncertainty

To further expand the model, we assume that individuals act in a world of uncertainty, where they adjust their expectations every period based on current and past information. The uncertainty can be accounted for via a forecasting error which incorporates past and future differences between realised values and their expectations.

Agents are assumed to form an expectation of wage adaptively based on past information. Due to the imperfection of forecasting, the wealth constraint needs to be updated every period. The updated wealth constraint in period 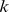, 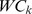, can be expressed as the sum between assets in period 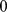, the discounted sum of total earnings between period 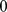 and period 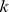, and the sum of expected total earnings, with discount factor 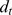 between period 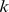 and retirement (period *T*): 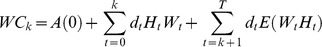



Alternatively, it can be expressed as the sum between assets in period 0, the expected discounted total earnings over the active life formed before entering the labour market and an adjustment error. The adjustment error equals the sum between the difference between realised earnings and their expectations made before entering the labour market over the period 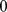 to 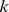 (part A), and the expectations adjustment of the total earnings made in period *t+1* to *T* (part B).
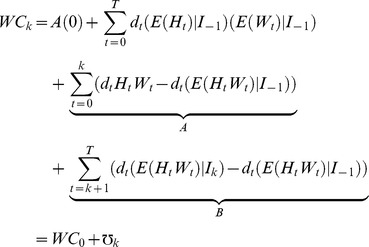




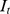
 reflects the information available to an agent in period *t* (e.g., personal and labour characteristics), and 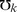 is the error adjustment term. Current expectations of lifetime wealth reflect past expectations and an error adjustment term that could either lower or raise the total constraint. To further simplify the equation, the interest rate is assumed certain. The error adjustment term 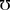 is expressed as:
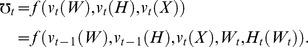




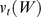
 is a vector of all wage information until period *t*, 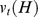 is a vector of working hours information until period *t*, and 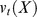 is a vector of personal characteristics until period *t*. As working hours are modelled using the wage rate and personal characteristics, the new adjusted lifetime wealth constraint can be proxyed by:
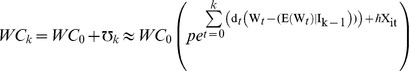




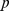
 is a scale parameter in the approximation, 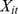 is a vector of personal characteristics which affect the form of the current expectation, and 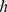 is the vector of coefficients of personal characteristics. Combining the anticipated paths for wages and income with the approximated empirical Lagrange function modifies the lifetime constraint. Therefore, 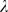 becomes: Å
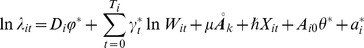





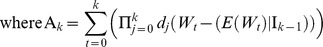




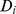
 is a vector of time-variant characteristics, 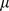 is an adjustment ratio, which shows the marginal change of working hours caused by the error term 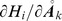. 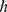 explains the role of personal characteristics in the expectation. It is not possible, however, to distinguish 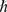 from the personal characteristics coefficient vector 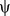 in equation (3).

## Empirical Implementation

This section introduces the econometric and the simulation techniques for estimating and simulating lifetime labour supply, both under certainty and uncertainty. The model is structured in three parts: a selection model, a model for lifetime wage profiles and a model for lifetime labour supply profiles.

### Data

We use the German Socio Economic Panel (GSOEP) data from 1996 to 2003 for estimation and 2004-2005 data for the simulation validation exercise. GSOEP is a nationally representative dataset of individuals living in Germany [21]. We consider only adult respondents below the retirement age (65). Table 1 presents basic employment statistics.

**Table 1:** Basic Descriptive Statistics of Employment Variables.

*Variable*	*Mean*	*Std. Dev.*
Age	40.52	12.78
Number of children	0.94	1.00
Employed in formal sector	0.50	0.50
Fulltime experience	1.63	1.49
Part-time experience	0.24	0.64
Unemployment period	0.39	0.79
Health situation	0.08	0.28
Hours of working	19.78	20.78
Education (Years)	11.43	2.95
Total number of observations	11456	

The dependent variable is the logarithmic value of actual working hours per week. The wage rate in the estimation and the simulation is calculated using actual working hours. “Participation” is defined as employment in the formal labour market, except for vocational training, zero working hours, military service and community service, which are all modelled as “non-participation” in the selection model.

### The Empirical Model of Lifetime Labour Supply under Certainty

This subsection develops the econometric model for the labour supply under certainty. The empirical model follows the structure of the economic model: the first model assumes homogenous preference parameters, and second one allows for a higher degree of heterogeneity amongst individuals.

#### An Econometric Model of Labour Supply under Certainty with Homogenous Preferences

For estimating a lifetime labour supply models which incorporates a lower degree of heterogeneity, we proposes a model similar to [22], [23]. The model extends the standard fixed effects specification to an estimation procedure which enables an efficient estimation of time-invariant and rarely changing variables by applying a “fixed effects vector decomposition”.

The lifetime labour supply model follows the structure of the economic model in (2):
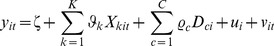
(4)



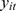
 is the natural logarithm of hours worked per week, 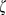 is the intercept of the base unit, *X* is a vector of time varying variables (natural logarithm of wage, age, age squared, children, health dummies, household type dummies, cumulated experience until last year (full time, part time, unemployment), other household income (only from women), sector dummies), *D* are time invariant variables (education, education squared, cohort dummies), 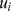 are the unobserved individual specific effects, 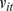 is an iid error term, and 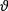 and 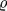 are parameters common to all individuals and constant over time.

The lifetime labour supply model is estimated using a three-stage procedure. To start, the model (4) is estimated using the standard fixed effects estimator and it is possible to extract the individual fixed effects or unit effects as:
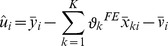



The estimated individual specific effect 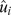 captures the unobserved individual-specific effect, the observed individual-specific effects *D*, the individual means of the residuals 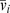 and the individual means of the time-varying variables. In the second step, the estimated individual specific effect is regressed against the observed time-invariant characteristics and the rarely changing variables to obtain the unexplained part of the individual-specific effects. The individual specific effects are decomposed as follows:
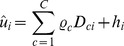
(5)



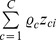
 is the explained part. 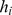 is the residual from equation (5) and captures the unexplained part of the individual-specific effect:
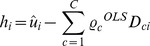



In the third stage, the individual effect from model (4) is substituted with the unexplained part of the decomposed individual fixed effect vector obtained in the previous stage, resulting in an error that is no longer correlated with the time varying covariates included in the model. Therefore, model (6) can be estimated consistently by pooled OLS:
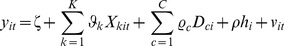
(6)


The Monte Carlo simulations conducted by [22] reveal the circumstances under which the FEVD is inferior to the pooled OLS, random effects (RE) and fixed effects (FE). OLS is more appropriate when there are no individual effects, RE when the individual effects are uncorrelated with the other explanatory variables, and FE when the assumptions of the RE are violated and the within-variance of the variables of interest is sufficiently large compared with the between-variance. If this condition does not occur, the vector decomposition technique has better finite sample properties in estimating models that have time-invariant or rarely changing variables correlated with individual-specific effects.

The lifetime wage process is estimated based on model (7) using the same procedure:
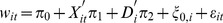
(7)



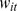
 is the natural logarithm of gross hourly wage is, 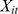 is a vector of time-varying variables, and 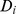 a vector of time invariant characteristics. 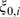 is the individual specific effect and 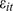 is the error term. The time-varying covariates in the wage equations are age, age squared, children, health dummies, household type dummies, cumulated unemployment experience until last year, and sector dummies. The time-invariant variables are education, education squared and cohort dummies.

When analysing the wage and the labour supply processes using panel data, the natural question that arises is whether the non-response or the missing observations are endogenously determined. One source of sample selectivity is the unbalanced nature of the panel. If the panel attrition is endogenous to the wage and the labour supply processes, sample selection would be informative for wage and hours, and therefore the estimates from the wage and hours equations would be biased. The panel attrition bias is disregarded from the analysis because previous studies using the GSOEP have indicated that the sample selection bias is not significant [24], [25]. The dataset is popular for analysing labour market issues.

Another sample selectivity bias comes from the fact that wages and working hours are observed only for the individuals in the labour market and the selection bias is determined by the differences between workers and non-workers. A selection bias arises when some component of the participation decision is relevant to the wage and hours processes. Disregarding these relationships, the estimates of wages and hours of work from the subsample of working individuals will be biased.

If the relationships between the participation decision and the wage and hours processes occur through the observables, the selection bias can be controlled by introducing the appropriate conditioning variables in the wage and hours equations. If the relationships between the participation decision and the wage and hours processes occur through the unobservable, meaning that the unobserved characteristics affecting the participation decision are correlated with the unobservable from the wages and hours equations, simply controlling for the observables is not enough to obtain unbiased estimates. If the observables are correlated with the unobservable, in order to get unbiased estimates, the wage and hours equations should include an estimate for the unobservable [26].

The selection model is defined using the latent variable model:
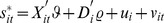



The selection indicator 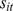 defines the observed employment status 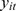:
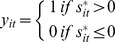



For fixed 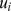 the probability of observing 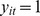 is given by: 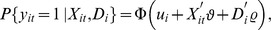




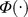
 is the cumulative distribution function and. 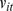 is assumed to be normally distributed. With i.i.d. error terms, the log likelihood function for a fixed effect probit model is given by: 
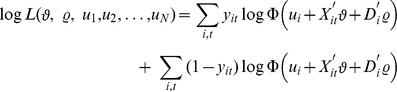



For a fixed *T* and 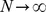, the maximum likelihood is inconsistent because the number of unknown parameters grows with the number of individuals in the sample. This is the so called “incidental parameter problem” which impedes the fixed effects probit model to be estimated consistently for a fixed number of periods. To circumvent this problem and to estimate the selection model accounting for the unobserved heterogeneity, the model decomposes the unobserved effect 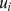, following the approach introduced by [27]. The assumption is that the unobserved effect can be modelled as:
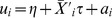
(8)


This equation assumes that the correlation between 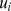 and 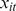 acts only through the time averages of the exogenous variables, whereas 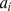 represents the remaining part of the unobserved effect which is independent of the time-varying variables. Equation (8) can be substituted into the selection model as follows:
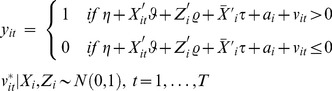
(9)


To summarize, the selection model includes time-invariant characteristics (education, education squared, cohort dummies, nationality dummies), time-varying variables (age, age squared, interaction between education and age, cumulated experience (full time, part-time, unemployment), health dummies, household type dummies, children and other household income (for women only)) and the means for the time-invariant variables (except for age).

To test for selection bias in both hours and wage equations for men and women, the study applied the fixed effect specification. For people participating in the labour market, the inverse Mills ratios were computed and then introduced into the initial models for hours and wages. The final step involved estimating the augmented models by applying the FE specification and testing the coefficient of the inverse Mills ratios. To correct for the sample selection in the wage and hours equation, the inverse Mills ratios for each year were included as time varying variables in the FEVD estimation of the wage and hours models.

For simulating both labour supply and wage processes, the main requirement imposed on the estimation method is to provide consistent and unbiased estimates. Allowing for unobserved heterogeneity and for a correlation between the unobserved individual effects and the other explanatory variables requires the use of FE estimation. This estimation technique, however, is not ideal in a simulation context given that FE cannot be simulated. An alternative is the FEVD technique, which circumvents the problems of a standard FE model (i.e. assumes a correlation structure between the unobserved individual effects and the other explanatory variables) by decomposing the individual effect and estimating the last stage as a pooled OLS.

#### An Econometric Model of Labour Supply under Certainty with Heterogeneous Preferences

For the estimation of the lifetime labour supply model which incorporates a high degree of individual heterogeneity, the study proposes a mixed fixed and random coefficient model similar to [28]. The fixed coefficients are considered constant across consumers and time, and the random coefficients are unique for each individual, but constant over time.

The assumption is that the labour supply model can be approximated by:
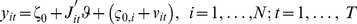
(10)where 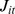 is 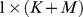
vector of explanatory variables, 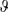 is the corresponding vector of coefficients, 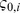 is the individual specific effect and 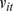 is the error term. The composite error term 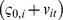 is correlated over time. Serially correlated errors imply then the OLS or maximum likelihood standard errors are no longer valid. This dependence can be taken into account either by using the sandwich estimator for the standard errors, which does not make any assumptions about the distribution of within-dependence of the residuals, or by modelling the dependence explicitly [28].

One way to model the dependence is to decompose the error component into a time-constant or a permanent error component 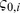, unique for each individual, and a transitory error component 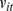, which varies across individuals and time. The permanent error component represents the combined effect of the omitted time-constant covariates and of the unobserved heterogeneity. 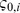 and 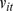 are assumed normally distributed, independent of each other. 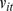 is assumed independent across individuals and over time. Model (10) can be rewritten by moving 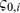 in the intercept:
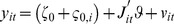
(11)


Model (11) represents a random-intercept model, which is a regression model with an individual specific intercept. 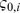 can be interpreted as a random parameter that is estimated together with the variance of the 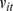. The parameters of the random-intercept model are estimated by maximum likelihood. To conclude, the linear random-intercept model allows the overall level of hours to vary across individuals after controlling for covariates.

Additional heterogeneity is incorporated by including additional random coefficients besides the random intercept, meaning that the effects of some covariates are allowed to vary across individuals. The resulting model is a mixed fixed and random coefficient model. By introducing individual-specific slopes, the assumption of parallel individual-specific regression lines is relaxed and our model becomes:
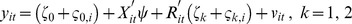




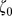
 represents the mean intercept of hours of work, 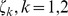 the mean slopes of the covariates chosen to have random coefficients, and 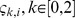 the deviation of individual intercept and slopes from the mean values of 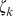.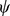 is a vector of fixed coefficients, constant across individuals and time. 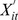 represent 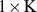 vector of covariates with fixed coefficients, whereas 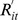, is 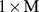 vector of covariates with random coefficients.

The wage model follows a similar specification; besides the random individual-specific intercept, the model is specified with two additional random coefficients for age and education:
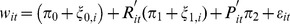




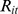
 is the vector the covariates estimated with random coefficients. 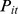 is the vector of covariates with fixed coefficients. 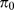 is the mean intercept of wage, and 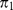 is the vector of the mean slopes of the variables 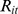. 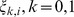 represents the deviation of individual intercept and slopes from the mean values of the point estimators 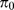, 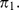


Given the high computation costs of estimating the selection model using a random coefficient specification, only fixed coefficient specification is used in the study. The inverse Mills ratios from the estimated probit regressions are included in the random coefficients wage equation of both men and women, whereas for hours, the additional term is included only for women.

### The Empirical Model of Lifetime Labour Supply under Uncertainty

The extension of the empirical model of lifetime labour supply to incorporate uncertainty is straightforward. The methodology is the same above, except for an additional regressor that captures the forecasting error. This term is introduced to incorporate that individuals adapt their expectations regarding their future wages each period.

#### An Econometric Model of Labour Supply under Uncertainty with Homogenous Preferences

Under uncertainty, the labour supply model assuming homogenous preferences is expressed as: 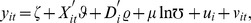


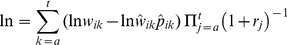
(12)



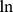
 represents the forecasting error equal to the cumulated discounted difference between the actual wage and the expected wage multiplied by the probability of being employed, from the start of the active life (*a*) until the current year. 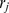 is the interest rate at time *j*. The selection and the wage models are the same as under certainty, assuming homogenous preferences.

#### An Econometric Model of Labour Supply under Uncertainty with Heterogeneous Preferences

The heterogeneous preference model under uncertainty follows the same procedure as the homogenous preference model under uncertainty, with the exception that the forecasting error in equation (12) has a random coefficient. This change implies that the effects of the variables vary across individuals, but the selection and wage models remain the same as under certainty. Table 2 summaries the differences among all proposed models.

**Table 2:** Overview of Proposed Models.

*Models*	*Estimation Method for Labour Supply*	*Uncertainty in Future Wages*
Standard Random Effect (Heckman extended) Model	Random Effects Model	No
Proposed Labour supply model with homogenous preferences	FEVD	No
Proposed Labour supply model with heterogeneous preferences	Mixed Coefficients Model	No
Proposed Labour supply model with homogenous preferences with uncertain extension	FEVD	Yes
Proposed Labour supply model with heterogeneous preferences with uncertain extension	Mixed Coefficients Model	Yes

All variables included in the estimations are the same except for the adjustment variable under uncertainty.

### Simulations

This section describes how the models are applied to simulate the labour supply responses for 2004 and 2005. The accuracy of the projections obtained from these models is compared with the simple extended Heckman model that is commonly used in simulating continuous labour supply. Since the GSOEP dataset is a panel, the Heckman model is extended to incorporate the unobserved heterogeneity. While the estimation of the probit model is identical with one used for the selection model, the second step of the Heckman procedure is estimated by a standard RE model.

**Figure 1:**
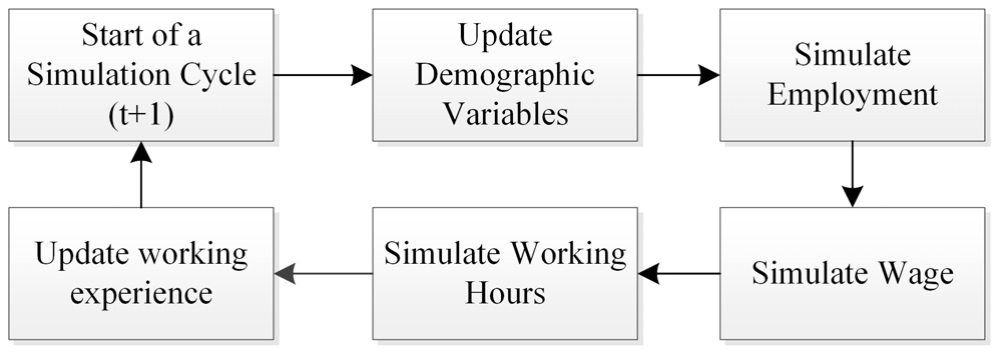
An Overview of Simulation Steps.

The simulations are performed using the estimates from the empirical models presented in the previous sections. The simulation follows the basic structure of a dynamic microsimulation. To simplify the exercise, this simulation only consists of the demographic and labour market module. The demographic module updates some basic demographic variables (e.g., age) over time. It also updates the variables that may interact with the demographic variables. The labour market module is the core part of the simulation, which updates the employment status, the wage, and hours of work. Figure 1 illustrates the steps of simulation.

For the employment model (selection model), the simulation uses pooled probit as yearly probit is unfeasible in a simulation exercise. This also circumvents the problem that the inverse Mills ratios cannot be updated during the simulation. The number of formally employed individuals is aligned with the real data in 2004 and 2005. The wage and the hours simulations are based on the updated personal characteristics and are the result of the selection simulation including the selection correction. The results of the wage simulation are used in the working hours simulation.

The simulation has the following sequence. It starts by determining the value of demographic variables in the new time period and updates the related variables. For the personal characteristics that are not influenced by employment, the simulation uses the actual characteristics observed in 2004 and 2005. The simulation then moves on to the next step where labour market variables are simulated. It predicts the probability of employed given the employment selection model. Afterwards, wage is predicted using the estimated equations. With the wage and personal characteristics information, it is now possible to simulate the hours of work. The correction terms for the models with the uncertainty extension are calculated right after the predicted wage becomes available. Lastly, the simulation updates the variables that reflect the labour trajectory, such as working experience (full time, part-time, unemployed), etc. The model projections are compared with the actual observed hours of work for 2004 and 2005. Both the estimation and the simulation is implemented using Stata. Fixed and random effects models are estimated using the standard panel commands xtreg, xtmixed is used for random coefficient model, and the fixed effect vector decomposition is estimated using the codes from [22].

## Results

This section presents the estimation and the simulation results. All models, including wage and labour supply models are estimated separately for men and women.

### Estimation Results

The parameter estimates for the probit models are largely skipped as the estimates are in line with previous findings. Inverse Mill's ratios are included in the wage and labour supply models, with the exception of male labour supply equation where the inverse Mill's ratio is not significant using any estimation method. For the random coefficients models, a test is performed to verify whether the random intercept model is sufficient to capture the heterogeneity in the wage and hours estimation. The likelihood-ratio test suggests that the random coefficients model fits better than the simple random intercept model, both for wage and labour supply.

Tables 3 and 4 show the parameter estimates for the wage equations for women and men. Both model specifications are standard. The age effect is as expected for both men and women in all three models: a positive impact with decreasing marginal effects, showing that hourly wage has the standard humped-shaped age pattern. When estimated using FEVD, the wage profile of women shows a stronger curvature than for men. The larger coefficients of the linear and quadratic age variables show that the growth rate of wages is higher for women at younger ages, but the growth rate reduces more rapidly at later ages than for men. After the introduction of heterogeneity, the impact of age is reduced for women, whereas for men it is increased. At younger ages, however, both men and women have a similar curvature of the wage profile. The estimated rate of return to education differs quite a lot between men and women and between the three models. The model estimated with FEVD illustrates a positive return to education for women, whereas for the other models the return appears insignificant. For men, the returns appear to be negative in the FEVD variant model and insignificant in the other models.

**Table 3:** Wage Function Estimates for Women 1996–2003.

*Variable*	*Fixed Effect Vector Decomposition*		*Mixed Fixed and Random Coefficients*		*Random-effects GLS (Extended Heckman)*	
	*Coefficient*	*Std. Error*	*Coefficient*	*Std. Error*	*Coefficient*	*Std. Error*
**Age**	0.1668	0.0061	0.1389	0.0109	0.1548	0.0073
**Sd (Age)**			0.0193	0.0010		
**Age Squared**	−0.0016	0.0001	−0.0015	0.0001	−0.0015	0.0001
**Unemployment Experience**	−0.0942	0.0024	−0.0772	0.0058	−0.0764	0.0057
**Education-age interaction**	−0.0004	0.0001	0.0006	0.0003	0.0007	0.0003
**Children**	−0.0721	0.0062	−0.0748	0.0104	−0.0698	0.0104
**Control for Health**	Yes		Yes		Yes	
**Control for Sector**	Yes		Yes		Yes	
**Control for Nationality**	Yes		Yes		Yes	
**Control for Year Effect**	Yes		Yes		Yes	
**Inverse Mills Ratio**	0.0694	0.0089	−0.0069	0.0131	−0.0126	0.0131
**Education**	0.0237	0.0104	−0.0337	0.0337	−0.0294	0.0311
**Sd (Education)**			0.0334	0.0094		
**Education Squared**	0.0022	0.0004	0.0028	0.0012	0.0024	0.0011
**Control for Cohort Effect**	Yes					
**Constant**			1.4544	0.3782		
**Sd (Constant)**			1.1352	0.0789		
**Number of observations**			14251		14251	
**Number of groups**			2767		2767	
**R^2^** **(overall)**					0.2969	

**Table 4:** Wage Function Estimates for Men 1996–2003.

*Variable*	*Fixed Effect Vector Decomposition*		*Mixed Fixed and Random Coefficients*		*Random-effects GLS (Extended Heckman)*	
	*Coefficient*	*Std. Error*	*Coefficient*	*Std. Error*	*Coefficient*	*Std. Error*
**Age**	0.1177	0.0044	0.1393	0.0082	0.1563	0.0059
**Sd (Age)**			0.0202	0.0009		
**Age Squared**	−0.0015	0.0000	−0.0015	0.0001	−0.0014	0.0001
**Unemployment Experience**	−0.0973	0.0022	−0.0705	0.0056	−0.0694	0.0055
**Education-age interaction**	0.0023	0.0001	0.0013	0.0003	0.0012	0.0002
**Children**	−0.0304	0.0046	−0.0154	0.0078	−0.0159	0.0078
**Control for Health**	Yes		Yes		Yes	
**Control for Sector**	Yes		Yes		Yes	
**Control for Nationality**	Yes		Yes		Yes	
**Control for Year Effect**	Yes		Yes		Yes	
**Inverse Mills Ratio**	0.0503	0.0126	0.0191	0.0168	0.0078	0.0167
**Education**	−0.1228	0.0084	−0.0831	0.0306	−0.0728	0.0288
**Sd (Education)**			0.0242	0.0120		
**Education Squared**	0.0026	0.0003	0.0029	0.0011	0.0026	0.0011
**Control for Cohort Effect**	Yes		Yes		Yes	
**Constant**			1.7804	0.2929		
**Sd(Constant)**			0.8657	0.0735		
**Number of observations**	17484		17484		17484	
**Number of groups**	2999		2999		2999	
**R^2^** **(overall)**	0.7793				0.3753	

Unemployment experience has a significant negative impact on wages for both men and women. The model with FEVD method illustrates the highest absolute impact, while the random coefficient model and the RE model show similar effects. In all three models, having or not having children influences wages negatively, and the impact in absolute value is higher for women than for men. The estimates of the inverse Mills ratios imply small positive correlations between the individual-specific error components of the selection model and the wage equation for men in all three models. For women, these correlations are negative in the mixed and the extended Heckman model.

To conclude, the estimation results from the wage models are in line with our expectations. Among all the specifications, the FEVD estimator appears to fit the data the best. The estimation results for the labour supply models are extensively examined in this study. Tables 5 and 6 present the coefficients estimated. Five labour supply models for both men and women are compared: the FEVD under certainty and uncertainty, the mixed fixed and random coefficients model under certainty and uncertainty, and the extended Heckman under certainty. The estimated coefficients are in general significant and stable across models.

**Table 5:** Hours Function Estimates for Women 1996–2003.

*Variable*	*Fixed Effect Vector Decomposition*				*Mixed Fixed and Random Coefficients*				*Extended Heckman*	
			
	*Certainty*		*Uncertain*		*Certainty*		*Uncertain*		*Certainty*	
	*Coeff.*	*Std. Error*	*Coeff.*	*Std. Error*	*Coeff.*	*Std. Error*	*Coeff.*	*Std. Error*	*Coeff.*	*Std. Error*
**Wage**	−0.2765	0.0051	−0.2742	0.0063	−0.1989	0.0151	−0.1998	0.0158	−0.2396	0.0082
**(Random Coeff sd)**					0.5259	0.0131	0.5286	0.0136		
**Age**	0.0367	0.0057	0.0267	0.0058	0.0019	0.0159	0.0061	0.0156	0.1174	0.0077
**(Random Coeff sd)**					0.0343	0.0011	0.0339	0.0011		
**Age Squared**	−0.0008	0.0001	−0.0007	0.0001	−0.0005	0.0001	−0.0005	0.0001	−0.0005	0.0001
**Experience Full time**	0.0164	0.0005	0.0184	0.0005	0.0365	0.0021	0.0365	0.0021	0.0305	0.0017
**Experience Part-time**	0.0359	0.0008	0.0327	0.0007	0.0239	0.0027	0.0200	0.0028	0.0141	0.0022
**Experience unemployment**	−0.1101	0.0024	−0.1269	0.0024	−0.0089	0.0069	−0.0145	0.0073	−0.0280	0.0061
**Education age interaction**	0.0012	0.0001	0.0008	0.0001	0.0007	0.0004	0.0003	0.0005	0.0009	0.0003
**Have child**	−0.0682	0.0156	−0.0850	0.0162	−0.0512	0.0200	−0.0665	0.0212	−0.0710	0.0230
**Other household income**	−0.0126	0.0010	−0.0124	0.0011	−0.0089	0.0011	−0.0080	0.0011	−0.0142	0.0014
**Education level**	−0.0050	0.0097	−0.0062	0.0100	−0.0488	0.0418	−0.0594	0.0431	−0.0755	0.0339
**Education Squared**	0.0003	0.0003	0.0007	0.0004	0.0029	0.0015	0.0036	0.0015	0.0030	0.0012
**Inverse Mills Ratio**	0.0684	0.0099	0.0068	0.0095	−0.0071	0.0122	−0.0218	0.0129	−0.0219	0.0140
**Wage Expectation Correction**			−0.0001	0.0000			0.0000	0.0000		
**(Random Coeff sd)**							0.0008	0.0001		
**Intercept (Random sd)**					2.5242	0.0678	2.5155	0.0732		
**Control for Health**	Yes	Yes	Yes	Yes	Yes	Yes	Yes	Yes	Yes	Yes
**Control for Sector**	Yes	Yes	Yes	Yes	Yes	Yes	Yes	Yes	Yes	Yes
**Control for Year effect**	Yes	Yes	Yes	Yes	Yes	Yes	Yes	Yes	Yes	Yes
**Control for Household Type**	Yes	Yes	Yes	Yes	Yes	Yes	Yes	Yes	Yes	Yes
**Control for Cohort Effect**	Yes	Yes	Yes	Yes	Yes	Yes	Yes	Yes	Yes	Yes

**Table 6:** Hours Function Estimates for Men 1996–2003.

*Variable*	*Fixed Effect Vector Decomposition*				*Mixed Fixed and Random Coefficients*				*Extended Heckman*	
			
	*Certainty*		*Uncertain*		*Certainty*		*Uncertain*		*Certainty*	
	*Coeff.*	*Std. Error*	*Coeff.*	*Std. Error*	*Coeff.*	*Std. Error*	*Coeff.*	*Std. Error*	*Coeff.*	*Std. Error*
**Wage**	−0.3351	0.0033	−0.3330	0.0035	−0.2099	0.0090	−0.2218	0.0092	−0.2796	0.0052
**(Random Coeff sd)**					0.3471	0.0071	0.3435	0.0073		
**Age**	0.1235	0.0031	0.1200	0.0031	0.0520	0.0053	0.0512	0.0054	0.1583	0.0040
**(Random Coeff sd)**					0.0173	0.0005	0.0178	0.0005		
**Age Squared**	−0.0008	0.0000	−0.0007	0.0000	−0.0006	0.0000	−0.0006	0.0000	−0.0008	0.0000
**Experience Full time**	−0.0633	0.0007	−0.0634	0.0007	−0.0028	0.0015	−0.0018	0.0016	−0.0033	0.0017
**Experience Part-time**	−0.0726	0.0012	−0.0799	0.0013	−0.0296	0.0030	−0.0278	0.0032	−0.0501	0.0035
**Experience unemployment**	−0.1323	0.0015	−0.1354	0.0016	−0.0185	0.0034	−0.0134	0.0035	−0.0423	0.0039
**Education age interaction**	0.0011	0.0001	0.0010	0.0001	0.0009	0.0002	0.0010	0.0002	0.0012	0.0002
**Have child**	0.0189	0.0116	0.0281	0.0117	0.0169	0.0146	0.0100	0.0152	0.0196	0.0174
**Other household income**										
**Education level**	−0.1214	0.0056	−0.1479	0.0058	−0.0500	0.0174	−0.0596	0.0184	−0.1058	0.0189
**Education Squared**	0.0013	0.0002	0.0025	0.0002	0.0012	0.0006	0.0015	0.0006	0.0025	0.0007
**Wage Expectation Correction**			−0.0002	0.0000			0.0000	0.0000		
**(Random Coeff sd)**							0.0005	0.0000		
**Intercept (Random sd)**					1.5028	0.0346	1.4760	0.0361		
**Control for Health**	Yes	Yes	Yes	Yes	Yes	Yes	Yes	Yes	Yes	Yes
**Control for Sector**	Yes	Yes	Yes	Yes	Yes	Yes	Yes	Yes	Yes	Yes
**Control for Year effect**	Yes	Yes	Yes	Yes	Yes	Yes	Yes	Yes	Yes	Yes
**Control for Household Type**	Yes	Yes	Yes	Yes	Yes	Yes	Yes	Yes	Yes	Yes
**Control for Cohort Effect**	Yes	Yes	Yes	Yes	Yes	Yes	Yes	Yes	Yes	Yes

The estimated wage elasticity is highly significant and stable for both men and women across models. Men record on average, in absolute value, a higher elasticity than women do. The highest wage elasticity is found under certainty when estimated using the FEVD. Assuming that the effect of wage varies across individuals dampens the magnitude of the effect. Assuming uncertainty, the effect increases by 2 percentage points. The Heckman model provides higher wage elasticity estimates than the random coefficient model.

As expected, the wage expectation correction has a small but significant negative effect on labour supply in both the FEVD and the random coefficient models. Incorporating that people adapt their expectations in a heterogeneous manner dampens the average impact of the wage expectation correction.

With regard to age, the labour supply profile of men illustrates a higher growth rate of hours worked at younger ages than the profile for women. The rate of growth in hours worked, however, reduces more rapidly toward later ages for men than for women. When the age effect is allowed to vary across individuals, its effect reduces in magnitude compared to the FEVD variant. The Heckman model provides the highest estimates for the age effect.

The estimates for the return to experience (full time and part-time) are similar across models: all key variables are significant, have the same shape and similar magnitudes. For men, the cumulated work experience has a negative effect on the lifetime labour supply response, which can be explained by the age-labour supply profile. For women, the cumulated work experience has a small positive effect on the labour supply response. Unemployment experience has a negative effect for the labour supply of both men and women, and the effect is higher in absolute value for women.

The presence of children in the family has a negative effect on the labour supply of women, whereas for men the effect is not significant. The effects are stable across models. For women, the estimates of the inverse Mills ratios differ greatly across models. The estimates of the Heckman model are similar to the random coefficient model under uncertainty. To conclude, the estimates are stable across the different model specifications and show on average a high level of significance. The FEVD model fits the best.

### Simulation Results

The simulation exercise evaluates the model's out-of-sample prediction performance. Each model is estimated on the same dataset and is used to predict labour supply in years 2004 to 2005. The simulation uses a simple dynamic microsimulation framework, where only crucial demographic variables are updated. The performance of the simulation is judged according to the distance between the actual value and the predicted value. Table 7 gives a general overview of each model's performance using this indicator. Both simulated and actual values are coded in logarithm scale.

**Table 7:** Simulation Models Comparison.

*Simulation Residual (logarithm)*	*Mean*	*Std. Dev.*	*Min*	*Max*
Heckman Extension Model	−0.354	0.432	−3.728	0.732
FEVD Fix coefficient with certainty	−0.027	0.444	−3.673	2.020
FEVD Fix coefficient with uncertainty	−0.031	0.441	−3.626	1.988
Mixed coefficients with certainty	−0.039	0.888	−8.364	8.936
Mixed coefficients with uncertainty	0.465	5.744	−50.933	55.689

The errors in the simulation come from three sources. The first source is the selection model, common to all models. The second source is the continuous labour supply model, which covers five different model setups. The last source is the wage estimation, as in the models under uncertainty the residual of the wage equation is crucial. In general, the FEVD fixed coefficients under certainty and uncertainty perform the best in terms of mean value and standard deviation of the simulated residuals. In terms of unbiasedness, the two FEVD estimated models and the mixed coefficients model under certainty perform better than the Heckman extension model. The mixed coefficients estimation, however, can be significantly affected by outliers and has a large standard error for the random intercepts, which may lead to errors in the simulation.

**Figure 2:**
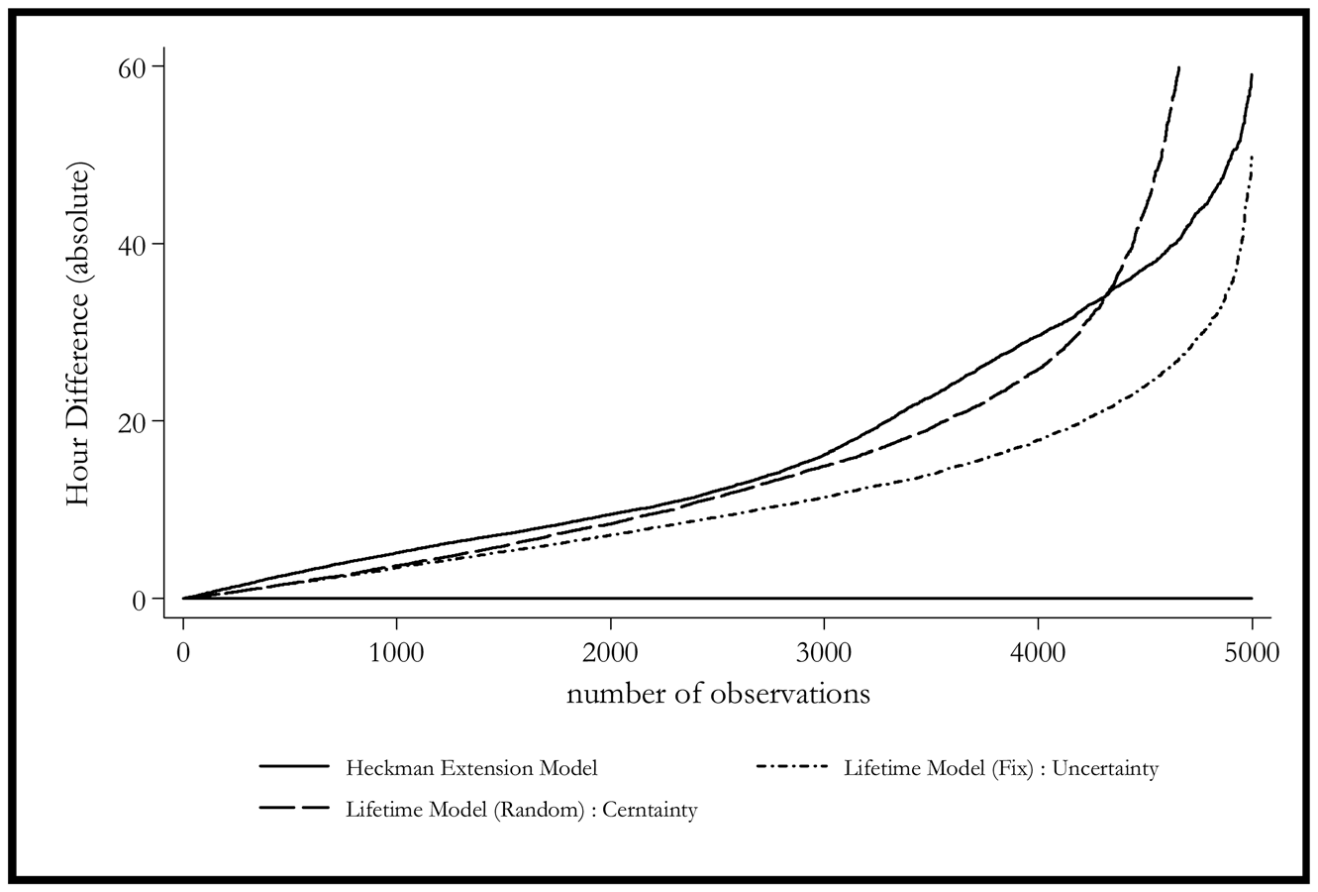
Simulation Residuals – Selected Models (2004–2005). olid Line: Heckman Selection Model. Dash Line: Lifetime Model (Random Coefficient): Certainty. Dash-Dot Line: Lifetime Model (Fix Coefficient): Uncertainty.

Figure 2 sorts the observations in terms of the absolute difference between simulated and actual values. Since the wage expectation adjustment (uncertainty) term does not play a major role numerically as suggested in the estimation result, the models with the uncertainty correction have a very similar curve compared with the ones without, provided the same estimation method is used. As a result, only 3 models are selected in Figure 2. The figure shows that the models estimated with the FEVD method performs the best in terms of the percentage of observations with the prediction error of less than 20 hours, while the Heckman extended model performs the worst judged by this criteria. This suggests that in a simulation study where the absolute error is a key factor, the proposed models should be estimated with FEVD.

**Figure 3:**
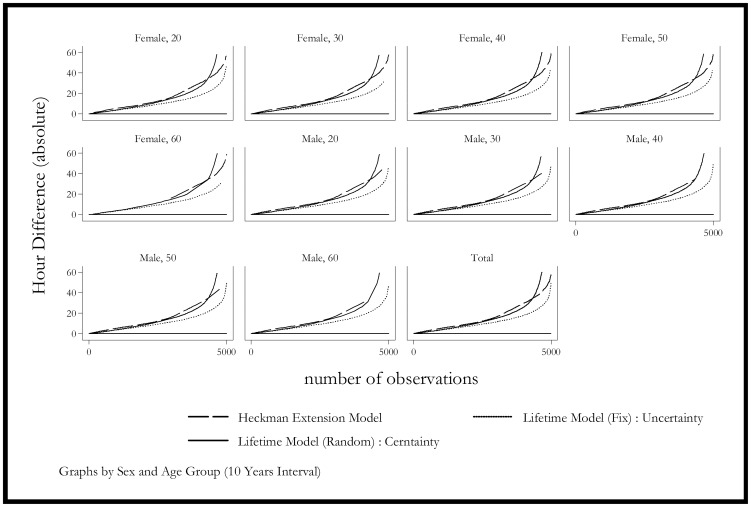
Simulation Residuals by Sex and Age - Selected Models (2004–2005). Dash Line: Heckman Selection Model. Solid Line: Lifetime Model (Random Coefficient): Certainty. Dot Line: Lifetime Model (Fix Coefficient): Uncertainty.

**Figure 4:**
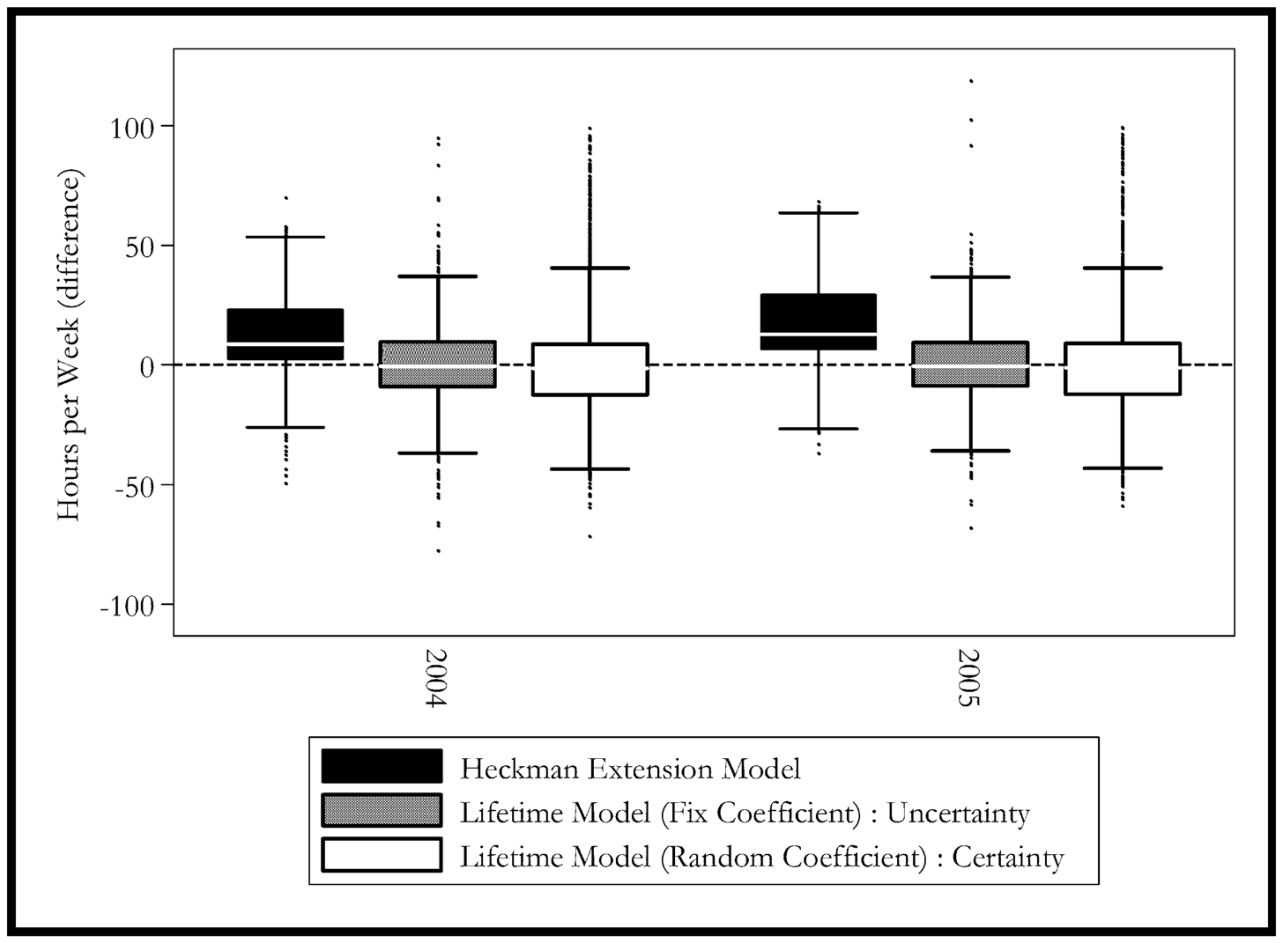
Simulated Hours Distributions by Year. Black Box: Heckman Selection Model. Grey Box: Lifetime Model (Random Coefficient): Certainty. White Box: Lifetime Model (Fix Coefficient): Uncertainty.

**Figure 5:**
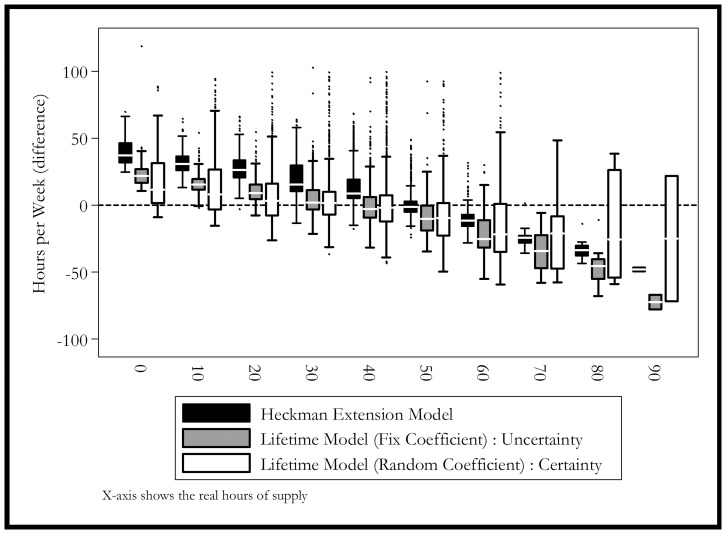
Simulated Distributions by Real Values of Working Hours. Black Box: Heckman Selection Model. Grey Box: Lifetime Model (Random Coefficient): Certainty. White Box: Lifetime Model (Fix Coefficient): Uncertainty.

Figure 3 extends the previous graph by showing the results for different groups, whereas Figures 4 and 5 show the distribution of the simulation for different groups. The simulation shows that the Heckman model is biased for 2004 and it continues to get worse in 2005. This bias may come from the biased estimation of time-invariant characteristics. The model estimated with mixed coefficients, although the mean predicted value is closer to the actual ones, it has a much larger standard error in the prediction. This finding is consistent with Figure 5, which shows that the mixed coefficients method has one of the most unbiased performances.

Overall, the FEVD variant of the model has the best simulation result according to the validation tests. The uncertainty extension, however, does not seem to have a large impact, this may partially be due to the error from the wage estimation. The mixed coefficient estimation seems to handle the heterogeneity better than the other models, but is less than ideal for simulations.

## Conclusion

This paper develops a structural lifetime model for estimating and simulating continuous labour supply. The model is consistent with the lifetime economic theory and is able to capture the individual heterogeneity to a larger extent than existing labour supply models by using more refined estimation techniques, including fixed effect vector decomposition (FEVD) and the mixed coefficients estimation method. In addition, one variant of the model loosens the certainty assumption in life cycle modelling. Instead, individuals are assumed to adjust their labour supply behaviour based on the differences between expected and actual earnings.

The paper compares different combinations of the model specifications and estimation methods, as well as the standard random effects model (Heckman) for their simulation performances. In a simple simulation presented in this paper, models were estimated with different combinations of estimation techniques and uncertainty correction term. The results suggest that the models estimated using the FEVD method has the highest prediction accuracy judged by the mean simulation error.

While the expectation correction is significant in the estimation, it is found to be less important in the simulation due to its relatively small coefficient and the potential wage estimation errors. When estimated using the FEVD method, the lifetime labour supply model developed in this study outperforms the Heckman panel extension model in the simulation, and this consistent across all calculated indicators. In practice, the models presented in this study could benefit the microsimulation models where continuous labour supply models are used.
